# Heterogeneity of endometriosis lesions requires individualisation of diagnosis and treatment and a different approach to research and evidence based medicine

**Published:** 2019-03

**Authors:** PR Koninckx, A Ussia, L Adamyan, A Wattiez, V Gomel, DC Martin

**Affiliations:** Latifa Hospital, Dubai, United Arab Emirates;; Professor emeritus OBGYN, KULeuven Belgium, University of Oxford-Hon Consultant, UK, University Cattolica, Roma, Moscow State Univ.;; Gruppo Italo Belga, Villa Del Rosario Rome Italy;; Consultant Università Cattolica, Roma Italy;; Department of Operative Gynecology, Federal State Budget Institution V. I. Kulakov Research Centre for Obstetrics, Gynecology, and Perinatology, Ministry of Health of the Russian Federation, Moscow, Russia; and e Department of Reproductive Medicine and Surgery, Moscow State University of Medicine and Dentistry, Moscow, Russia;; Professor Department of obstetrics and gynaecology, University of Strasbourg;; Professor emeritus Department of Obstetrics and Gynecology, University of British Columbia and Women’s Hospital, Vancouver, BC, Canada;; Professor emeritus School of Medicine, University of Tennessee Health Science Centre, Memphis Tennessee, USA; Institutional Review Board, Virginia Commonwealth University, Richmond, Virginia. USA.

**Keywords:** endometriosis, heterogeneity, evidence based medicine, medical treatment, statistics

## Abstract

Statistical significance is used to analyse research findings and is together with biased free trials the cornerstone of evidence based medicine. However traditional statistics are based on the assumption that the population investigated is homogeneous without smaller hidden subgroups.

The clinical, inflammatory, immunological, biochemical, histochemical and genetic-epigenetic heterogeneity of similar looking endometriosis lesions is a challenge for research and for diagnosis and treatment of endometriosis. The conclusions obtained by statistical testing of the entire group are not necessarily valid for subgroups. The importance is illustrated by the fact that a treatment with a beneficial effect in 80% of women but with exactly the same but opposite effect, worsening the disease in 20%, remains statistically highly significant.

Since traditional statistics are unable to detect hidden subgroups, new approaches are mandatory. For diagnosis and treatment it is suggested to visualise individual data and to pay specific attention to the extremes of an analysis. For research it is important to integrate clinical, biochemical and histochemical data with molecular biological pathways and genetic-epigenetic analysis of the lesions.

## Statistics and evidence in non-homogeneous populations

**Figure 1:**
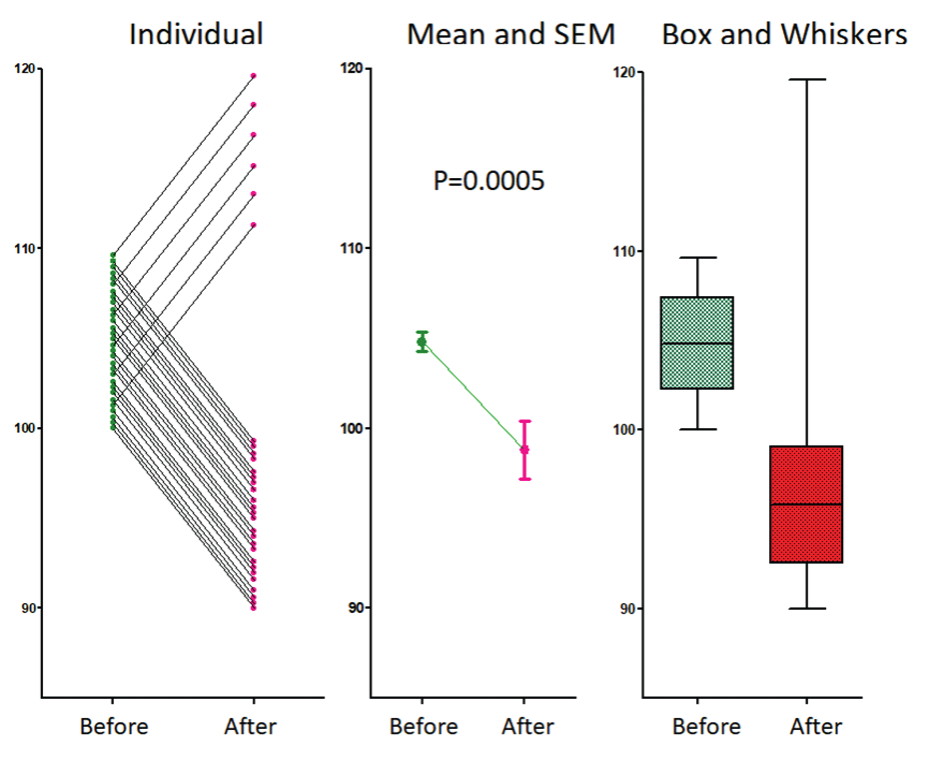
A data set with 24 women decreasing and 6 increasing pain by 10% after treatment. Heterogeneity of response is obvious when the individual data are plotted but hidden when only means and SEM are given. The variability in response is suggested by box and whiskers plots. Students paired and un-paired t tests and Mann-Whitney results in P=0.0009, P=0.0004 and 0.0001 respectively.

Evidence based medicine (Djulbegovic and Guyatt, 2017) requires that diagnosis and treatment are based on the best evidence available and on statistical significance. However, traditional statistics to demonstrate specificity and sensitivity of a diagnostic test and efficacy of a treatment are based on the assumption that the population investigated is homogeneous without hidden subgroups. Conclusions and significances are valid only when this condition is met (Connor, 2015; Reinhart, 2015). Homogeneity of the population with a disease is assumed in most studies until subgroups with a different behaviour are discovered. Figure 1 illustrates how easily statistical significances can hide an obvious sub-group.

A treatment with a 10% decrease in 24 women in symptoms, and a 10% increase in 6 women. This opposite effect in 20% of women, is obvious when individual data are plotted. However, this is hidden with means and SD while the t-test remains highly significant, whether performed as a paired or unpaired test for a normal distribution or a Mann-Whitney test for a non-normal distribution. Visualisation of data with Box and whiskers plots could lead us to suspect that something is wrong. That traditional statistical tests do not detect hidden subgroups is well known (Farland et al., 2016). Moreover, the difficulty to detect hidden subgroups increases when the prevalence of a different behaviour or the differences in effect are less.

In most publications on endometriosis, a traditional statistical analysis is used to evaluate endometriosis data or the evaluation of the efficacy of a therapy. This is questionable since the basic assumptions of a homogeneous and normally distributed population are rarely met given the heterogeneity of endometriosis lesions (Farland et al., 2016).

## Endometriosis is a heterogeneous disease

Endometriosis is macroscopically a heterogeneous disease varying from subtle to typical, cystic and deep lesions. More important is that macroscopically similar looking lesions can cause very different symptoms and can have a different behaviour. Pain symptoms poorly correlate with the severity of lesions. Deep endometriosis is associated with severe pain in most women, but occasionally some lesions do not cause pain. During pregnancy, most endometriosis lesions will decidualize and regress (Koninckx et al., 2018), but occasionally deep lesions progress and cause bowel perforations (Setubal et al., 2014). Only some deep endometriosis lesions have cancer-associated driver mutations (Lac et al., 2019). The inflammatory reaction around macroscopically similar lesions is variable. The associated changes in plasma and in peritoneal fluid are highly variable and characterised by large standard deviations. The aromatase activity and progesterone resistance in the lesions vary from inexistent to very pronounced (Bulun et al., 2015; Koninckx et al., 2019). Although most endometriosis lesions require estrogens to grow, occasionally lesions can develop in the absence of significant estrogen concentrations in blood as observed in men and in rare cases of deep endometriosis nodules developing more than 10 years after menopause in the absence of estrogen intake (Asencio et al., 2018). The clinical effect or progestogen therapy on endometriosis associated pain varies from pronounced to no effect (Vercellini et al., 2018).

We recently proposed the genetic-epigenetic theory (Koninckx et al., 2019) which can explain the variability of similar looking lesions in symptoms and in biochemical changes in lesions and in plasma. This theory also explains the occurrence of endometriosis in the absence of endometrium, the clonal and the hereditary aspect of endometriosis lesions and all known associated observations.

We are born with a variable set of genetic and epigenetic incidents, some transmitted from our parents at conception, and some occurring during pregnancy. After birth additional genetic-epigenetic incidents occur. They can be caused by accidents during cell division, by environmental toxicity, by oxidative stress or by radiation. Taking into account the redundancy of intracellular pathways (Leonard and Lin, 2000; Stoney et al., 2018), we suggested that endometriosis lesions start to develop when the cumulative set of incidents reach a certain threshold. The further growth and development of each lesion is determined by their specific set of incidents and their environment such as the peritoneal cavity, and the inflammatory and immunologic reaction. In addition, the estrogen stimulated growth, and the subsequent shedding and bleeding in endometriosis lesions constitute a repetitive tissue trauma and repair (ReTIAR). This causes an inflammatory reaction, and an oxidative stress, which might favourise additional genetic-epigenetic incidents in the lesion (Suda et al., 2018). The role of local cell-cell interactions and the effect of critical mass and immunology are not yet understood. Clinically, the genetic-epigenetic theory thus postulates that the set of genetic and epigenetic incidents at birth explains the hereditary predisposition to develop endometriosis, and many of the associated differences such as biochemical changes in the endometrium, the infertility, the changes in pregnancy and the immunologic changes.

## The clinical consequence of heterogeneity is individualisation of therapy

In the absence of markers to diagnose the different types of endometriosis lesions, the indications of heterogeneity of similar looking lesions are strong enough to rethink our management of women with endometriosis. The absence of data that permit to predict the natural history of a lesion, hampers decisions of treatment in younger women. Early surgery might prevent the subsequent growth, but risks to surgically treat a transient finding with no long-term benefit. That the well-known delay in diagnosis can be problematic in growing lesions, emphasises early diagnosis and strict follow-up. Also, prevention of progression should comprise the prevention of growth and the prevention of additional genetic or epigenetic incidents, by reducing environmental pollution and by reducingthe oxidative stress caused by repetitive (abundant retrograde) menstruation and by bleedings in the lesions. The role of anti-oxidants and food intake is unclear but might be considered.

The realisation that medical treatment can have a variable effect (Koninckx, et al., 2018; Vercellini et al., 2018) and does not stop growth in all lesions, suggest a strict follow-up with monitoring of growth when given for many years. Treatment should be reconsidered when associated with growth or incomplete pain relief. However, it is unclear whether ultrasound or other non-invasive methods permit to monitor growth and how women with a moderate pain reduction only should be judged. Anyway it cannot be taken for granted that oral contraception will prevent progression in all women. It seems logical that lesions with a marked progesterone resistance will rather be stimulated than inhibited by oral contraceptives.

Individualisation of surgery today is based on past experience and common sense (Koninckx et al., 2017). We do not know when complete excision of deep endometriosis is required and when a less aggressive excision is sufficient. For cystic ovarian and bowel endometriosis, surgery balances completeness of surgery, organ damage and recurrence rates. It is accepted that non-growing deep endometriosis lesion without clinical symptoms should not be treated. It is not clear whether surgery should be performed before proceeding to IVF in these women. The risks of progression during pregnancy and the risk of stimulating growth by puncture during IVF pick-up are unpredictable.

Age is another important variable and after menopause the ovarian cancer risk strongly suggest surgery in symptomatic endometriosis. In order to tailor surgery we should know whether the peripheral cells of a lesion are abnormal endometriotic cells requiring a complete excision (Garcia-Solares et al., 2018) or whether these cells are a reversible metaplasia induced by the endometriotic cells, making a less aggressive excision sufficient. The same applies to the treatment of cystic ovarian endometriosis which balances between completeness of surgery, ovarian damage and recurrence rates.

## Research should be adapted to a heterogeneous disease

The knowledge that endometriosis is heterogeneous should stimulate to look more carefully at individual data and at the effects of therapy over time. Visualisation of different populations might be useful also for clinical symptoms and biochemical data, as progesterone resistance or aromatase activity which seem a continuum from very little to very strong. The statistical analyses as developed for redundant pathways (Stoney et al., 2018) and Bayesian statistics which are more intuitive, could be more appropriate for heterogeneous populations.The need for a disease stratification of endometriosis beyond macroscopical inspection requires the integrative analysis of genomic, epigenomic and phenotypic data. This suggests a focus shift from larger series, to more complete data. However, the combination of clinical and biochemical data in plasma and in the lesions together with molecular biological pathways requires the co-operation between groups with very different expertises (Becker et al., 2014). In addition, the multivariate analysis might require prohibitively large groups.

As clinicians, we know that historically many discoveries in medicine were made by investigating accidents of nature such as androgen resistance. Applied to endometriosis, this suggests that the investigation of rare events and of outliers should become more important. Examples are endometriosis lesions growing in the absence of circulating estrogens after menopause (Asencio et al., 2018), deep endometriosis lesions progressing during pregnancy (Setubal et al., 2014) deep endometriosis lesions without pain, and the ‘strange’ endometriosis lesions occasionally seen during surgery. The latter comprise fast progressing lesions, highly proliferative deep endometriosis lesions, and cystic ovarian endometriosis with abundant ‘endometrium like’ tissue. For medical therapy it is suggested to investigate the extreme responses which varies from strong to no response.

## Conclusions

In conclusion, the clinical, inflammatory, immunological, biochemical, histochemical and genetic-epigenetic heterogeneity of similar looking endometriosis lesions is a challenge for research, diagnosis and treatment of endometriosis. Traditional statistics which are based on homogeneous populations to calculate significances are inappropriate to detect hidden subgroups as recognised before (Farland et al., 2016).

As clinicians we suggest focussing investigation on the extremes and to exploit the clinically rare cases which might be informative as ‘accidents of nature’. This requires that the investigation of unpredictable rare events is organised through collaboration between surgeons and research groups of varying expertise.

Heterogeneity of endometriosis should also be reflected in publications with more Scatchard plots of individual data and their changes over time, instead of the limited information provided by means, standard deviations and P values. 
